# Timing predictability enhances regularity encoding in the human subcortical auditory pathway

**DOI:** 10.1038/srep37405

**Published:** 2016-11-17

**Authors:** Natàlia Gorina-Careta, Katarzyna Zarnowiec, Jordi Costa-Faidella, Carles Escera

**Affiliations:** 1 Institute of Neurosciences, University of Barcelona, P. Vall d’Hebron 171, 08035, Barcelona, Catalonia, Spain; 2 Brainlab – Cognitive Neuroscience Research Group, Department of Clinical Psychology and Psychobiology, University of Barcelona, P. Vall d’Hebron 171, 08035, Barcelona, Catalonia, Spain; 3 Institut de Recerca Sant Joan de Déu, Santa Rosa 39-57, 08950, Esplugues de Llobregat, Catalonia, Spain

## Abstract

The encoding of temporal regularities is a critical property of the auditory system, as short-term neural representations of environmental statistics serve to auditory object formation and detection of potentially relevant novel stimuli. A putative neural mechanism underlying regularity encoding is repetition suppression, the reduction of neural activity to repeated stimulation. Although repetitive stimulation per se has shown to reduce auditory neural activity in animal cortical and subcortical levels and in the human cerebral cortex, other factors such as timing may influence the encoding of statistical regularities. This study was set out to investigate whether temporal predictability in the ongoing auditory input modulates repetition suppression in subcortical stages of the auditory processing hierarchy. Human auditory frequency–following responses (FFR) were recorded to a repeating consonant–vowel stimuli (/wa/) delivered in temporally predictable and unpredictable conditions. FFR amplitude was attenuated by repetition independently of temporal predictability, yet we observed an accentuated suppression when the incoming stimulation was temporally predictable. These findings support the view that regularity encoding spans across the auditory hierarchy and point to temporal predictability as a modulatory factor of regularity encoding in early stages of the auditory pathway.

The encoding of regularities in the acoustic environment appears as a critical mechanism for auditory perception, as regularities shape perceptual objects in complex auditory scenes^1^,^2^. Short–term predictive representations of acoustic regularities are derived from the probability of occurrence of repeating events, so that computed statistical regularities serve as a basis to automatically detect deviant events which do not match such predictions^3^,^4^.

Regularity encoding has been inferred by studies on deviance detection^5^,^6^, in which low–probability (“deviant”) sounds are presented amongst high–probability (“standard”) sounds. A more direct approach has been taken in studies measuring repetition suppression (RS), the attenuation of neural responses to repeated stimulation^7^,^8^, which proposed RS as a potential mechanism underlying regularity encoding^9^ and therefore, sensory memory-trace formation^10^,^11^.

In the auditory modality, regularity encoding has been shown in human auditory cortex, as demonstrated by the modulation by probability of long– and middle– latency auditory evoked potentials^10^,^12^,^13^,^14^,^15^,^16^,^17^ and functional magnetic resonance imaging (fMRI)^18^, as well as in subcortical auditory stages as revealed by fMRI^19^,^20^. Compelling evidence is provided by animal studies of single unit recordings, which have disclosed stimulus–specific adaptation in primary auditory cortex^21^,^22^,^23^ and in auditory subcortical stations, including the inferior colliculus^22^,^24^ and the medial geniculate complex of the thalamus^25^,^26^.

From a predictive coding account, it has been suggested that RS reflects the correct prediction of the upcoming stimulus, that is, a reduction of the prediction error for expected stimuli. This model emphasizes the importance of contextual factors, such as the probability of a stimulus repetition^27^, or the temporal predictability of the upcoming stimulus on the encoding of regularities. Yet, temporal predictability of the auditory input has been shown to shape predictions in auditory cortical areas, as stimuli occurring at predictable temporal intervals advance the onset of repetition positivity, a brain potential correlate of RS, when comparing predictable to unpredictable stimulus presentations^9^,^28^.

The present study was designed to ascertain whether the modulation of RS by temporal predictability could be present in subcortical stages of auditory processing. For that aim, we measured the Frequency Following Response (FFR)^29^, a sustained component of the auditory brainstem potential that is phased-locked to the periodic characteristics of the eliciting stimulus. The FFR is highly sensitive to context-dependent contingencies^30^,^31^ and to real-time statistical properties of the stimulus^29^,^32^, and has been used to show regularity encoding and deviance detection in human auditory brainstem^33^,^34^. Hence, we hypothesize that FFR will be modulated both by stimulus statistics and temporal predictability, revealing that even early neural representations of sound are sharpened by temporal expectation of the statistical regularities.

## Results

To assess temporal predictability effects on regularity encoding on the FFR, stimulus were delivered in two timing conditions. In the Predictable timing condition, stimuli were presented with a constant stimulus onset asynchrony, thus allowing a temporal prediction of the occurrence of the upcoming stimulus. In the Unpredictable timing condition, stimuli were presented with a jittered stimulus onset asynchrony so that the temporality of the upcoming stimulus could not be anticipated.

**Figure 1:**
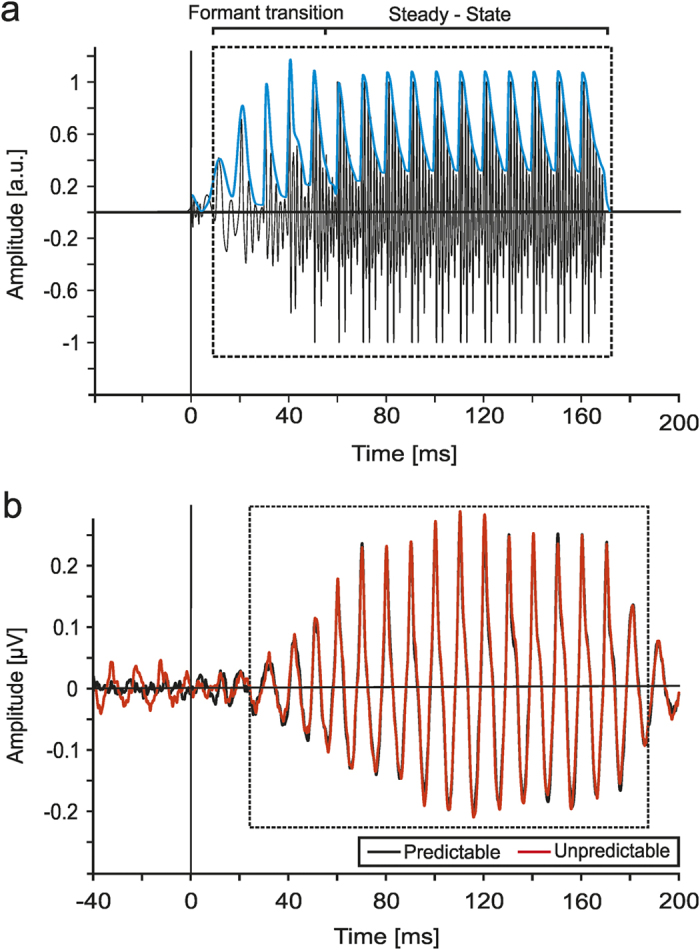
Stimulus waveform and Frequency Following Responses elicited in the two temporal conditions. (**a**) The acoustic waveform of the stimulus/wa/. The envelope of the stimulus is highlighted in blue. The formant transition region and the vowel steady–state region are bracketed (a.u. = arbitrary units) (**b**) Grand-average FFR response recorded at Cz of all participants in the predictable (black) and unpredictable (red) timing conditions recorded to the/wa/stimuli presented against a continuous babbling background noise. As can be seen here, the envelope of the stimulus (**a**, blue) was preserved in the response (**b**) of both timing conditions. This is evidenced by the framed areas, which include the same number of cycles.

The grand–average waveforms of FFRs elicited to both Predictable and Unpredictable timing conditions are depicted in Fig. 1b. As expected, the waveforms of both timing conditions resembled markedly the stimulus envelope (Fig. 1a), and a small difference in the response between both timing conditions can be seen. Below we describe in detail the influence of timing predictability and the effects of repetition in these auditory subcortical responses.

**Figure 2:**
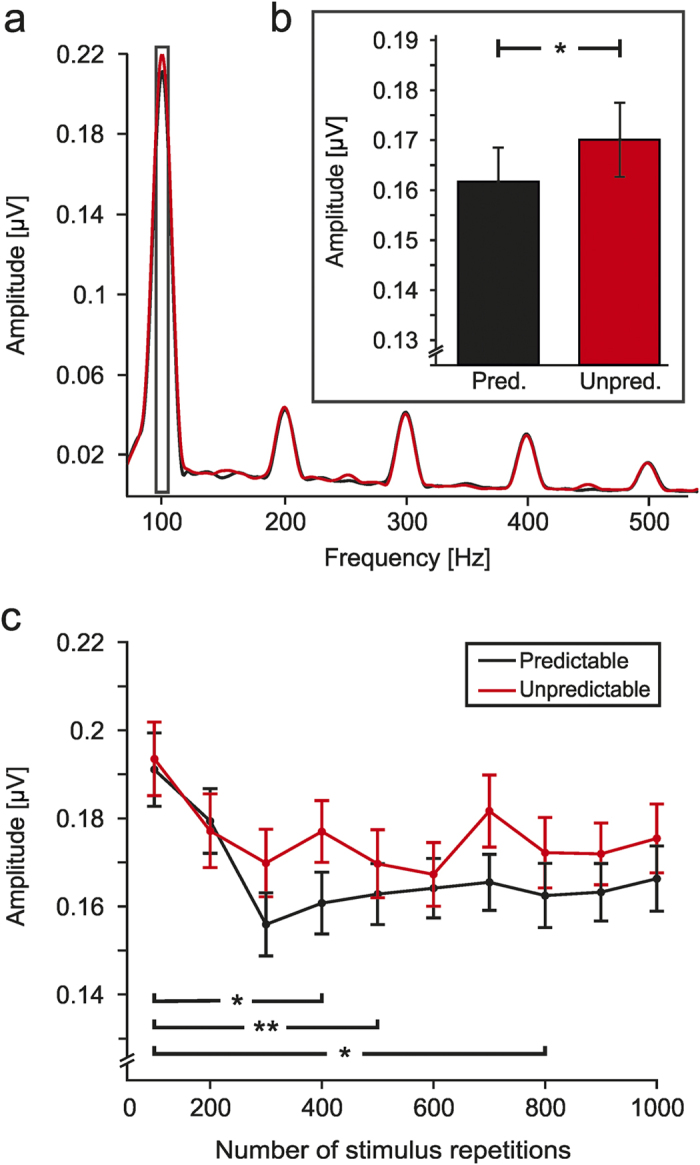
FFR amplitude spectrum and mean amplitude of the fundamental frequency peak. (**a**) FFR amplitude spectrum of the steady–state part of the response in the Predictable (black) and Unpredictable (red) timing conditions. (**b**) Mean amplitude of the F0 (100 Hz), computed over a 20 Hz window around the peak, is represented for both conditions. The Unpredictable timing condition yielded significantly larger amplitudes than the Predictable condition. Pred = Predictable; Unpred = Unpredictable. (**c**) Mean spectral amplitude of the F0 at ten consecutive 100–epoch sub–averages in both Predictable (black) and Unpredictable (red) timing conditions. Decreased amplitude was observed in the Predictable condition compared to the Unpredictable timing condition. Also, a decrease in amplitude was observed as the number of previous repetitions increases in both timing conditions. Error bars represent ±1 SEM. Statistically significant comparisons are marked with one (p < 0.05) or two (p < 0.01) asterisks.

When analysing the timing predictability effects of the auditory sequence on the neural response, FFRs showed a significant effect for Condition (F(1,29) = 5.091, p = 0.032, 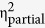 = 0.149; Fig. 2a,b). The neural response to the incoming sounds had a larger amplitude when the timing was unpredictable (mean = 0.17 μV, SE = 0.08 μV) compared to when the same stimuli were presented in a predictable manner (mean = 0.16 μV, SE = 0.07 μV), thus indicating enhanced adaptation to timing–predictable repetition.

Moreover, after averaging the responses to analyse the effects of stimulus repetition across time (Fig. 2c), larger FFR amplitudes were found for the Unpredictable (mean = 0.176 μV, SE = 0.014 μV) compared to Predictable timing condition (mean = 0.167 μV, SE = 0.013 μV; Condition: F(1,29) = 5.649, p = 0.024, 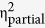 = 0.163). Repetition effects were also statistically significant (Repetition; F(9,261) = 3.832, p < 0.001, 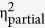 = 0.117), indicating a decrease in the FFR amplitude as the stimulus history increased, for both timing conditions. Further post-hoc paired t-tests between repetition–averages in both conditions revealed a significant repetition effect between sub–averages 1–100 and 301–400 (t(29) = 3.673, p = 0.043), 1–100 and 401–500 (t(29) = 5.157, p = 0.001) and 1–100 and 701–800 (t(29) = 3.609, p = 0.049). There were no further significant differences in F0 amplitude between the remaining positions. The interaction between timing predictability and repetition did not reach statistical significance (Condition x Repetition: F(9,261) = 0.684, p = 0.724, 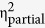 = 0.023).

**Figure 3:**
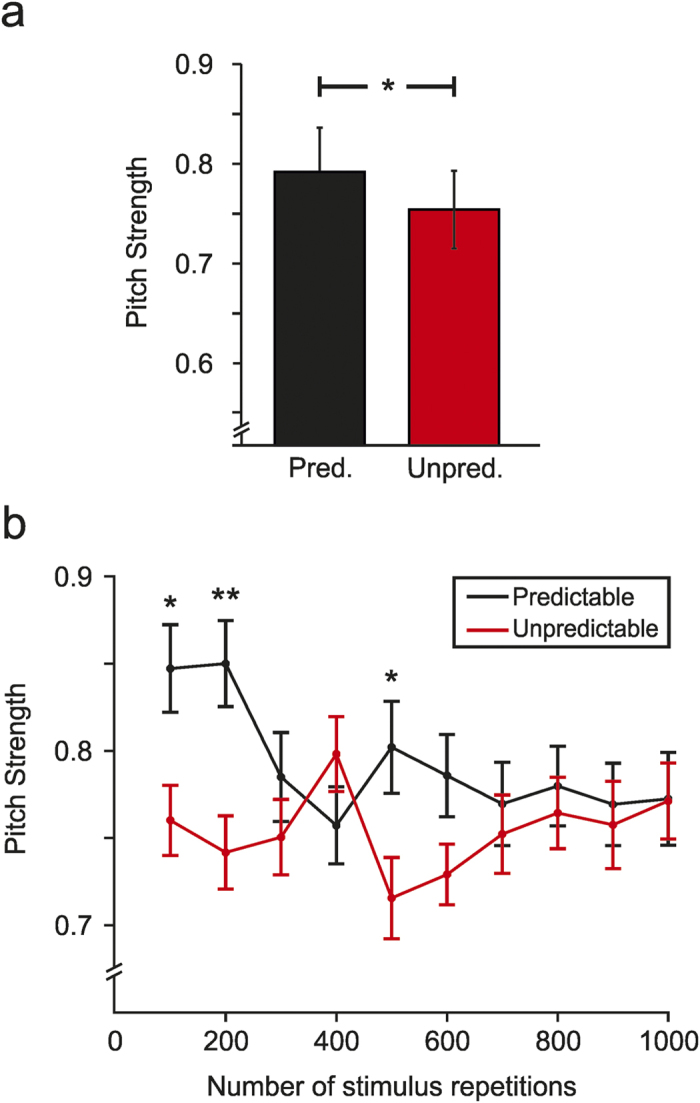
Neural Pitch Strength to the pitch of the stimulus waveform in both timing conditions. (**a**) Pitch strength Fisher transformed correlation values in the Predictable (black) and Unpredictable (red) timing conditions. Increased phase–locking to the stimulus F_0_ was observed on the Predictable compared to the Unpredictable timing condition. Pred = Predictable; Unpred = Unpredictable (**b**) Pitch strength Fisher transformed correlation values at ten consecutive 100–epoch sub–averages in both Predictable (black) and Unpredictable (red) timing conditions. Different trends can be distinguished for both conditions as the number of repetitions increased. Error bars represent ±1 SEM. Statistically significant comparisons are marked with one (p < 0.05) or two (p < 0.01) asterisks.

Pitch strength values indicated a stronger phase-locking to the stimulus F0 contour when the timing was predictable (mean = 0.792, SE = 0.045) compared to when the stimuli were presented in an unpredictable manner (mean = 0.754, SE = 0.04; Condition: F(1,29) = 8.122, p = 0.008, 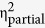 = 0.219; Fig. 3a). Furthermore, Pitch strength showed separable patterns in the two timing conditions across history of repetitions (Condition × Repetition: F(9,261) = 2.807, p = 0.004, 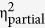 = 0.088; Fig. 3b). When stimuli occurred with an unpredictable timing, the encoding of the overall periodicity of the signal did not change as the number of repetitions increased. However, when the stimuli were presented in a predictable manner, the initial phase-locking to the stimulus was very high, but as the number of repetitions increased, the pitch strength values decreased to the same level as the unpredictable timing condition values. Further post-hoc analysis indicated that Pitch strength values differed between conditions on sub–averages ranging 1–100 (t(29) = 2.709, p = 0.011), 101–200 (t(29) = 4.307, p < 0.001) and 401–500 (t(29) = 2.462, p = 0.02).

## Discussion

The present study constitutes the first demonstration that temporal predictability enhances regularity encoding of the repetitive acoustic environment in the human auditory subcortical pathway. In particular, we have shown that the reduction of neural response caused by repetitive stimulation, although present independently of temporal aspects of the auditory input, is in fact modulated in the subcortical auditory system by the temporal predictability of the incoming stimulus. Indeed, we found a decrease in FFR amplitude when the auditory stimuli were presented with a constant presentation rate compared to when these very same stimuli were delivered at random time intervals, precluding the precise temporal anticipation of their occurrence. In addition, a general decrease on the FFR amplitude was observed as the history of stimulation increased. This effect on the FFR amplitude was clearly observed for both timing conditions, thus indicating that independently of the temporal context of the auditory stimulation, the FFR is suppressed when it faces a repetitive acoustic stimulus. Interestingly, the modulatory effects of the temporal aspects of the acoustic input on the FFR amplitude became evident only after the accumulation of 200 stimuli repetitions, when the suppression caused by the repetitions reached a plateau, causing an enhancement on the suppression when the stimuli were temporally predictable.

Our findings favour the importance of timing as a key factor in the encoding of acoustic regularities and the formation of stimulus–specific memory traces along the whole auditory hierarchy. Temporal predictability of the incoming auditory stimulation has been shown to reduce the amplitude of the P50^35^ and N1 components^35^,^36^ of the auditory evoked potentials, and to enhance both repetition suppression^28^,^37^ and the repetition positivity^9^ in human auditory cortex, and has been suggested to boost the propagation of regularity encoding upstream the auditory pathway^8^,^9^. In this regard, our results expand previous findings on the role of temporal predictability on regularity encoding, by disclosing the sensitivity of the subcortical auditory pathway to temporal predictability, thus supporting the view that the mechanisms that govern regularity encoding at cortical levels also expand to subcortical stages^5^.

Interestingly, the effect of the temporal predictability on the subcortical auditory system that we are describing here appears as an enhancement of the repetition suppression, that is, as a pronounced reduction of the neural response to the repetitive stimulation^7^. Previous findings on animal studies established repetition suppression as a phenomenon that expands along the auditory hierarchy. By means of single cell recordings in anesthetized animals, it has been shown that individual neurons at both cortical^21^,^22^,^23^ and subcortical^24^,^25^,^38^ levels exhibit a reduced response to a stimulus that is presented repeatedly. Repetition suppression has also been observed in the animal cortical auditory steady state responses (ASSR), as an amplitude habituation of this periodic electrical brain oscillation evoked by sinusoidally modulated acoustic stimuli^39^. In agreement with these animal findings at subcortical level, a recent human study described that when a stimulus feature (e.g., pitch) is repeated, the blood oxygen level–dependent (BOLD) activity can be either reduced or enhanced^19^, thus revealing that repetition suppression is a phenomenon that is not exclusive of the auditory cortex but that it can be also observed at lower stages of the auditory hierarchy. Our data confirm and expand these findings, as well as the observations from animal studies, agreeing with the emerging view that regularity encoding is a property that spans the whole auditory anatomical hierarchy, from the brainstem upwards, and in multiple temporal dimensions^5^,^6^,^13^,^15^.

The observed sharpening of the neural representations by temporal predictability is in line with hierarchical predictive coding models^40^,^41^,^42^,^43^. These posit two functionally distinct subpopulations of neurons, one to encode the expectations of perceptual inputs and one for the prediction error. According to these models, the predictive population builds up an internal model of the regularities within the incoming stimulation in order to form relevant predictions, so that predictions at different levels of the processing hierarchy try to explain away the prediction error on preceding levels. At the same time, the predictive error population compares the incoming input to the predictions encoded by the predictive populations of neurons. The activity of the prediction error population is transmitted to the predictive population as a feedback and this error signal is used to adjust the internal model. In this line, when the auditory input is temporally predictable, the input matches the prediction coming from upper levels, thus reducing the prediction error response. On the other hand, when the auditory stimulation is temporally unpredictable, there is a decrease on the prediction error due to the repetitive characteristics of the stimulation, but there is a mismatch on the temporal expectation, leading to a repetition suppression that it is not, however, as strong as the one produced by the temporally predictable stimulation. Although the FFR has been shown to be quite insensitive to higher order perceptual processes^44^, it is indeed modulated by stimulus regularities^30^,^33^,^34^,^45^, which indicates that the online formation of predictive models via stimulus regularity encoding is reflected at subcortical levels despite that already established categories to interpret acoustic stimulation may not require them.

Notably, our results provide two complementary views of the effects of temporal predictability on regularity encoding in the human subcortical auditory pathway. On one side, as described above, the observed decrease on the F0 amplitude, which reflects the neural suppression underlying the encoding of regularities on the subcortical auditory pathway, as well as its modulation by the temporal predictability of the upcoming sounds. On the other side, by capitalizing on the high faithfulness of the FFR to the incoming stimulus^29^, we observed that the modulation of the early representations of regular sounds by the temporal structure of the auditory input is partially due to an increase in the robustness of the phase-locking in the auditory subcortical structures, thus indicating that the temporal predictability of the incoming stimulation increases the signal to noise ratio of the encoded repetitive stimuli. Although both findings may seem contradictory, they are, in fact, complementary, as to the periodicity of the signal contributes not only the fundamental frequency but also the whole spectral richness of the response^46^. The increased pitch strength magnitude indicates that the response is more periodic and the phase-locking to the stimulus is more reliable^47^,^48^, thus helping the extraction of acoustic features. As the number of temporally predictable repetitions increases, the encoding of the stimulus periodicity is reduced, revealing that whilst new predictable stimulation facilitates the neural phase-locking to the stimulus, a repeated stimulation reduces the need to represent the stimulus in a fine-grained manner. This decrease goes in parallel to the adaptation we observed on the spectral domain, where the phenomenon of repetition suppression is well described. Interestingly, the increased neural phase-locking to the incoming repetitive stimulation helps the extraction of acoustic features and aids the subcortical auditory system to better encode the upcoming repetitive stimulation, thus making unnecessary for the auditory subcortical structures to respond strongly to the temporally predictable repetitive stimulus presentation. On the other hand, when stimuli were temporally unpredictable, there was a smaller neuronal phase-locking to the incoming stimulation but these values where stable as the stimulus history increased. Consequently, a suppression of the FFR amplitude is observed, as the stimuli are repetitive, but this suppression is reduced.

Taken together, these complementary findings led us to speculate that the temporal predictability of the upcoming stimulation may be influencing the encoding of regularities by helping the extraction of the important stimulation amongst a noisy environment. By means of this mechanism, the temporal predictability of the regular stimulation would help to extract all the features of the sounds and induce a better phase-locking of the subcortical structures to it. On the other hand, a non-temporally–predictable regular stimulation would not allow the subcortical structures to phase-lock to the auditory input as faithfully as when stimuli were predictably delivered, but as the history of stimulus presentation increases, the same early stages of the auditory hierarchy will keep extracting all the features possible from the sounds that are being presented, even if the neural response to those stimuli decreases.

In summary, our study has shown that temporal predictability modulates the auditory FFR to a repeated stimulation, leading to enhanced repetition suppression when the incoming auditory stimuli are temporally predictable compared to when the temporality of the following sound could not be predicted. Despite this enhancement on response suppression, a temporally predictable presentation aids the encoding of the presented sounds by increasing the signal to noise ratio. Altogether, we have demonstrated that early neural representations of sounds are sharpened by the temporality of the encoded statistical regularities. Our findings add to the evidence in favour of the back–propagation hypothesis^8^, which posits that with an increasing number of stimulus repetitions, a stimulus-specific memory trace can be detected earlier on the auditory hierarchy. This hypothesis was broadened when timing was proposed to be an important variable for the formation of the aforementioned memory trace at the level of the primary auditory cortex^9^. Crucially, our results support the view that timing is, indeed, a critical factor that affects the formation of the stimulus-specific memory trace along the whole auditory hierarchy.

## Methods

### Participants

Thirty paid university students (aged 19–27 years, mean age = 22.1 years, 8 males, 3 left–handed) with no history of auditory, neurological or psychiatric disorders participated in the study. All participants lived in a Catalan/Spanish-speaking environment and all but two (Basque and Polish) had Catalan, Spanish or both as their mother language. Hearing thresholds were assessed with a standard pure-tone audiometry at the beginning of the experimental session using Bayerdynamic DT48-A headphones (Bayerdynamic GmbH & Co, Heilbronn, Germany). Mean hearing thresholds were below 25 dB SPL for the five test frequencies (250, 500, 1000, 2000 and 4000 Hz) in all the participants. As music experience is known to modulate the encoding of the fundamental frequency (F0) of complex sounds at the level of the brainstem^49^, all participants were enrolled with less than 4 years of musical training that ceased five or more years before the study. The study was approved by the Ethical Committee of the University of Barcelona and was in accordance with the Code of Ethics of the World Medical Association (Declaration of Helsinki). Written informed consent was obtained from each participant before starting the experiment.

### Stimuli and procedure

The auditory sequence was composed of a consonant–vowel (CV) syllable/wa/33, generated with the Klatt speech synthesizer^50^. The syllable had a duration of 170 ms and a F0 of 100 Hz. Third (F3), fourth (F4), and fifth (F5) formants were set at 2900, 3500 and 4900 Hz respectively. In order to elicit a large onset response, the first 5 ms of the CV syllable consisted of a rapid glide in the first (F1; from 400 to 1700 Hz) and second (F2; from 1700 to 1240 Hz) formants. After the initial 5 ms, there was a 50 ms transition in F1 from 125 to 800 Hz and in F2 from 571 to 1200 Hz.

During the auditory stimulation with the CV syllable, a Spanish six–talker babble (four females and two males, 75 s track) was played as a background noise (10 dB SPL lower than the stimuli) in order to create a challenging listening situation^49^. To create the babble, speakers were recorded in a sound attenuated booth when reading in a comfortable and conversational manner semantically anomalous sentences. Tracks were acquired with 44 kHz sampling rate and 16-bit accuracy using Audacity 2.0.0 (Audacity Team® 2012). After offline root mean square amplitude normalization in Matlab v7.4 (Mathworks), all the recordings were circularly shifted and mixed together in such a way that the beginning of each speaker’s track was delayed 10 s in reference to the previous speaker recording. To assure that there was no interaction between the background noise and the/wa/stimulus, the babble was looped with no silent intervals during the experimental blocks and CV presentation was started at a random phase of the babble.

The/wa/stimuli were presented binaurally at 75 dB SPL in alternating polarities via ER-3A ABR insert earphones (Etymotic Research, Inc., Elk Grove Village, IL-USA) in two different timing conditions: Predictable and Unpredictable. In the Predictable timing condition, stimulus onset asynchrony (SOA) was set to 366 ms. In the Unpredictable timing condition stimuli were presented with a variable SOA jittered between 183 and 549 (mean SOA 366 ms) in seven equiprobable steps of 61 ms arranged randomly. Each condition was divided into 8 blocks, each block consisting of 1001 presentations of the/wa/stimulus. Blocks of the two conditions were presented alternately and the order was counterbalanced across participants.

During the experiment, participants sat comfortably in an electrically and acoustically shielded room and were instructed to relax and watch a silent subtitled movie of their choice, while ignoring the auditory stimulation. Pauses between blocks lasted 30 s, during which participants were allowed to move. Recording time lasted a total of two hours.

### EEG recording

FFRs were extracted from the continuous EEG recording acquired with Neuroscan 4.4 software and Neuroscan SynAmps RT amplifier (NeuroScan, Compumedics, Charlotte, NC, USA). The EEG was recorded from 36 scalp Ag/AgCl electrodes mounted in a nylon cap (Quick-Cap; Compumedics, Charlotte, NC, USA) at the standard 10–20 system locations. Two additional electrodes were positioned at the left and the right mastoids (M1 and M2, respectively). The electrooculogram (EOG) was measured with two bipolar electrodes placed above and below the left eye (VEOG), and two horizontal electrodes placed on the outer canthi of the eyes (HEOG). The ground electrode was located between Fz and FPz, and the right earlobe (A2) served as an online reference. All impedances were kept below 10 kΩ during the whole recording session and data was online bandpass–filtered from 0.05 to 3000 Hz and digitized with a sampling rate of 20 kHz.

### Data processing and analysis

Data analysis was performed offline using EEGlab v.7 toolbox^51^ running under Matlab v.2012a. The continuous recordings extracted from the Cz electrode were filtered offline with a bandpass Kaiser window FIR filter from 70 to 1500 Hz and epoched from 40 ms before the stimulus onset to 180 ms after the stimulus. Epochs for Predictable and Unpredictable timing conditions were sorted separately. Trials with activity greater than 35 μV were removed from any further analysis and remaining epochs were baseline corrected to a 40 ms interval preceding the sound onset^52^.

Data was averaged in two different manners. To analyse the effects of temporal predictability on the FFR, epochs from each timing condition and for each participant were averaged separately (Predictable condition: mean = 7746 trials, std = 283.7; Unpredictable condition: mean = 7730 trials, std = 342.7). To analyze the effects of stimulus repetition on the FFR across time, each experimental block was divided in ten consecutive runs, each containing 100 stimulus repetitions. For each participant and condition separately, each run was averaged with the corresponding one from the other experimental blocks of the same condition. This way, we could obtain an estimation of the response based on 1000 stimulus presentations to cumulative repetition (i.e., from 1–100, 101–200, 201–300, 301–400, 401–500, 501–600, 601–700, 701–800, 801–900 and 901–1000 repetitions) for each condition separately. After artifact rejection, in the Predictable condition, 772 trials were included on average on each 100–repetition sub–average (std = 4.37), and the Unpredictable condition consisted of 770 trials per 100–repetition sub–average (std = 5.49). Responses to alternating polarity stimuli were averaged together to minimize stimulus artefact and cochlear microphonic, preserving the FFR to the stimulus envelope^53^.

Only the steady-state part of the FFR was analysed (65–180 ms), as rapid formant transitions are a perceptual challenge for the auditory system^54^. Additionally, previous studies using the consonant-vowel stimulus/da/demonstrated that the FFR elicited by transition from the consonant to the vowel differ from the responses elicited by the steady-state vowel part of the stimulus^30^,^49^. Therefore, as the FFR encodes better the periodic part of the stimuli, we focused on the region of the response which corresponds to the vowel steady–state part.

Fast Fourier Transform (FFT)^55^ was applied to demeaned, zero-padded (1-Hz resolution) averages, windowed with a Hanning tapper. The mean response amplitude was computed using 20-Hz-wide window surrounding the F0 (90–110 Hz) and the subsequent five harmonics: H2, H3, H4, H5, and H6. These harmonic components were, however, not reliably present in all participants and therefore only response to the F0 was statistically analysed. Overall condition effects were assessed by means of repeated–measures ANOVA with the factor Condition (Predictable vs. Unpredictable); repetition effects in the two conditions were computed with repeated–measures ANOVAs with the factor Condition (Predictable vs. Unpredictable) and Repetition (ten 100-epoch sub–averages).

Neural Pitch Strength was quantified to analyse the magnitude of the neural phase-locking in the subcortical auditory pathway to the pitch of the stimulus waveform in both timing conditions. It was derived using a short-term autocorrelation analysis from 15 to 175 ms with 40–ms sliding window and a 1–ms step. This procedure involved cross–correlating a 40–ms frame of the response with itself and finding the height of the first peak in the autocorrelation function away from time-lag zero, which was taken as the magnitude of neural pitch strength^47^,^56^,^57^,^58^,^59^. In all cases, this peak fell at a time lag of approximately 10 ms, which corresponds to the fundamental pitch period of the stimulus (i.e., frequency = 1/periodicity; e.g., 100 Hz = 1/10 ms). To account for the transmission delay of the earphones and the neural delay, the analysis bin began at 15 ms for the responses. Pitch strength values obtained from each time frame of response were Fisher-transformed and averaged, resulting in one value per each 100–epoch sub–average (ten values in total). Pitch strength on the two timing conditions was analysed with repeated–measures ANOVA with the factor Condition (Predictable vs. Unpredictable) and Repetition (ten 100-epoch sub–averages).

The Greenhouse–Geisser correction was applied when the assumption of sphericity was violated, and results were corrected using the Bonferroni correction to adjust for multiple testing. Additional Bonferroni–corrected post–hoc tests were performed to examine the direction of the effects. Significance was defined for p ≤ 0.05.

## Additional Information

**How to cite this article**: Gorina-Careta, N. *et al.* Timing predictability enhances regularity encoding in the human subcortical auditory pathway. *Sci. Rep.* **6**, 37405; doi: 10.1038/srep37405 (2016).

**Publisher’s note:** Springer Nature remains neutral with regard to jurisdictional claims in published maps and institutional affiliations.
